# Smart Polymer/Carbon Nanotube Nanocomposites and Their Electrorheological Response

**DOI:** 10.3390/ma7053399

**Published:** 2014-04-30

**Authors:** Ke Zhang, Hyoung Jin Choi

**Affiliations:** 1 School of Chemical Engineering and Technology, Harbin Institute of Technology, Harbin 150001, Heilongjiang, China; 2 Department of Polymer Science and Engineering, Inha University, Incheon 402-751, Korea

**Keywords:** electrorheological fluid, core-shell, carbon nanotube, polymer nanocomposite

## Abstract

This review article summarizes the preparation of polymer/carbon nanotube (CNT) nanocomposites and their applications as electrorheological (ER) fluids. These ER fluids exhibited a controllable electro-response under an applied electric field due to the presence of well-dispersed CNTs. The background, morphology, preparations, and characteristics of these materials are discussed, specifically focusing on the various approaches in the preparation of polymer/CNT nanocomposites, morphology, and their effects on the ER characteristics.

## 1. Introduction

**Figure 1.**
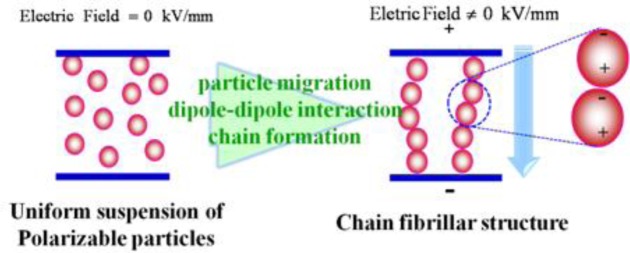
Schematic diagram of ER fluid system with and without an applied electric field.

Electrorheological (ER) fluids, which are considered smart and intelligent fluid systems, are generally composed of electrically-polarizable particles dispersed in an insulating liquid, whose state can be changed from a liquid-like to solid-like in the order of milliseconds under an applied electric field [1–7], as shown in Figure 1. This is because once an electric field is applied to an ER fluid, the field-induced dipoles in the dispersed particles attract each other, forming chains or fibrillated structures towards the field, resulting in sudden changes in the rheological and mechanical properties, which increase with increasing electric field strength. The transition of the properties of ER fluids is reversible so that after removing the electrical field, the fluid returns to the state where all particles are dispersed randomly within the medium phase. This interesting phenomenon under an applied electric field is similar to magnetorheological (MR) fluids when they are controlled by the application of an external magnetic field. Therefore, these unique properties give ER fluids potential applications in a range of engineering fields including engine mounts, torque transducers, shock absorbers, vibration attenuators, control systems, and ER polishing [8].

Although early studies on the ER materials focused on extrinsically polarizable particles, e.g., silica, alumina, mesoporous molecular sieves, starch, and cellulose, several problems of these hydrous ER fluids with a narrow operation temperature, device corrosion, water evaporation, and dispersion instability have limited their applicability in the operation. To overcome these impediments, anhydrous ER materials, which are typical conducting materials, have been developed, and include polyaniline (PANI) [9–13], poly(p-phenylene) [14,15], polypyrrole (PPY) [16–19], and PANI derivatives, e.g., PANI nanoparticles [20], PANI composite microbeads [21], and PANI core-shell structured particles [22–25].

Recently, CNTs have been introduced as a new candidate for the preparation of ER materials owing to their unique and versatile electrical properties. On the other hand, multi-walled carbon nanotube (MWNT) has been adopted more widely in this field than single-walled carbon nanotube (SWNT) because of their relatively low electrical conductivity avoiding electric short circuits under a high electric field applied for the ER test. Kozinsky and Marzari [26] characterized the response of isolated MWNTs and nanotube bundles to static electric fields using first-principles calculations and density functional theory. The longitudinal polarizability of the MWNT and nanotube bundles is given by the sum of the polarizabilities of the constituent tubes, whereas the transverse response in MWNTs is dominated by the outer few layers. Extensive studies on carbonaceous materials [27–29] and polymer/carbon family nanocomposites including carbon black, CNT, carbon fiber, and carbonized material, such as ER materials, have been reported [30–42]. In particular, ER materials were developed by depositing MWNTs on the particle surface to produce a core-shell structure [36–42]. In contrast, polymer/MWNT-based ER materials have been prepared via a range of polymerizations (dispersion polymerization [33], suspension polymerization [34,43], and emulsion polymerization [44]) in the presence of MWNTs. Owing to the dispersion of MWNT into a monomer prior to polymerization, some MWNTs pierce the microspheres to produce a “kebab-like” [34] or necklace structures [31] after polymerization. On the other hand, the as-produced MWNTs contain many impurities, such as metal catalyst and amorphous carbon, which can interfere with the desired properties of the CNTs. Acid and heat treatments are used widely to remove the impurities prior to use. After purification, there is a challenge in fabricating polymer/MWNT nanocomposites because the nanotubes aggregate easily to form bundles or ropes via van der Waals force, and they are difficult to disperse in most common solvents and monomer solutions. The introduction of various functional groups onto the MWNT surface, assistance with the surfactant or the use of a sonication method can help improve the dispersion in the medium. Concentrated acid solutions (HNO_3_, H_2_SO_4_ and their mixtures) are generally used to purify CNT by removing fullerenes and catalyst particles, and producing functionalized sites, such as carboxylic and hydroxyl on the CNT sidewall. During this oxidation process, only a part of the CNT structure is destroyed and grafted with functionalized groups on the defects. For example, MWNTs after oxidation were confirmed by TGA to be approximately 13 wt% of the functional groups attached to the CNT side wall due to the residual weight [45]. In addition, for poly(methyl methacrylate) (PMMA) grafting to MWNT, 25 wt% PMMA was found to be grafted onto the CNT side wall by TGA after removing the un-grafted PMMA [46]. Therefore, the grafting ratio of the functional groups on the side wall of CNT is not high. The introduction of functionalized groups onto the CNT side wall does not affect the mobility of the MWNTs and can improve the dispersibility of MWNTs.

This paper reviews the synthesis and characteristics of the polymer/MWNT nanocomposites used as ER materials that were prepared using a range of methods, such as non-covalent adsorption, *in situ* dispersion, suspension, and oxidative dispersion polymerization, with particular focus on the change in morphology and physical properties after adding the MWNTs. The ER performance of the polymer/MWNT nanocomposites was observed either by optical microscopy (OM) or rotational rheometry depending on the state of the nanotubes in the nanocomposites.

## 2. ER Materials

### 2.1. Core-Shell Structured ER Particles

Core-shell structured ER particles are made from micron-sized particles, such as PS, PMMA, and PMMA snowmen, with different shapes and diameters in the core wrapped by MWNTs. The “core materials” are generally insulating particles that can be synthesized using a range of polymerization techniques. The functionalized MWNTs act as “shell materials” that are carried out to coat the as-prepared particles to form a conducting layer on the particle surface not only by non-covalent adsorption but also by chemical covalent bonding. In this process, the diameter of the MWNTs should be 8–40 nm, which is much smaller than that of the particles used to prepare the ER particles [37]. Furthermore, the adsorption state of the MWNTs on the particle surface is strongly dependent on the dispersion medium [40], surfactant [38], or polyelectrolyte [41] used. The coating layers of the MWNTs not only provide polarizable moments on the particle surface, but also supply considerably rough surfaces that can induce surface friction in the dispersing medium.

#### 2.1.1. Non-Covalent Adsorption

**Figure 2.**
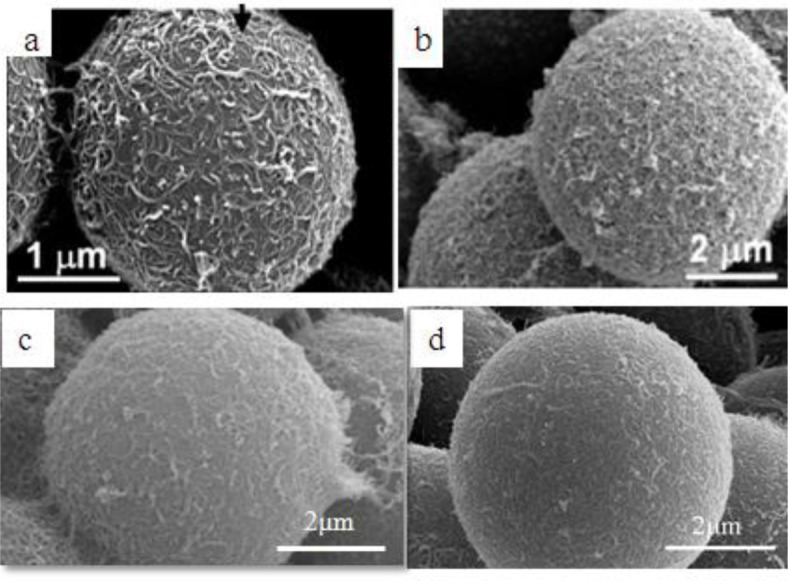
SEM images of the carbon nanotube-adsorbed (**a**) PS and (**b**) PMMA microspheres using the surfactants of CTAB [37]; (**c**) c-MWNT adsorbed PMMA microspheres obtained in Di-water [39]; (**d**) core shell structured MWNT-NH_2_/PMMA particles [40] (reprinted with permission from [38,40,41], Copyright 2005 American Chemical Society; 2011 Elsevier; 2012 Elsevier).

**Figure 3.**
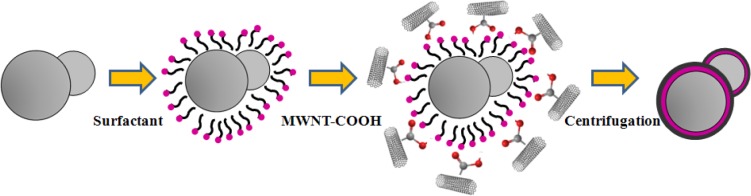
Preparation route for the c-MWNT–SL particles (reprinted with permission from [36], Copyright 2012 The Royal Society of Chemistry).

**Figure 4.**
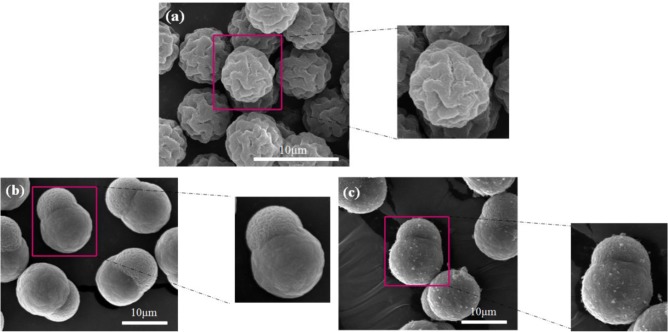
SEM images of (**a**) PMMA seeds; (**b**) pure SL particles; and (**c**) c-MWNT (0.96 wt%) adsorbed SL particles (reprinted with permission from [36], Copyright 2012 The Royal Society of Chemistry).

Surfactants, as additives and protecting agents, are used widely in nanomaterials synthesis to adjust the shapes and sizes of the nanocrystals [47,48] and other nanomaterial systems [49–51]. In particular, the surfactant, which was made up of a water soluble (hydrophilic) and water insoluble (hydrophobic) part, can be adsorbed at the interface between the immiscible bulk phases of the particles and solution in these MWNT/polymeric bead cases to reduce the surface tension. Jin *et al.* [37] fabricated MWNTs-adsorbed polystyrene (PS) and PMMA microspheres using a range of surfactants, such as anionic sodium dodecyl sulfate (SDS) and sodium dodecylbenzene sulfonate (NaDDBS), cationic cetyl trimethylammonium bromide (CTAB), and nonionic Triton X-100, respectively. In the experiment, the dispersion of PMMA (6.5 μm) and PS (3.0 μm) microspheres were dropped sequentially into a glass bath containing the nanotube (after acid treatment) dispersion with different types of surfactants using a syringe pump. With time, the settled portion of the MWNTs-adsorbed PMMA microspheres was obtained at the bottom of the glass vials. The product was washed several times with pure water to remove the remaining surfactants and dried in a vacuum oven at room temperature. SEM (Figure 2a,b) revealed the best result when the MWNTs were dispersed in an aqueous solution of CTAB, showing a densely-dispersed MWNT coating on the particle surface. The 3.9 wt% MWNTs in the MWNTs-adsorbed PMMA microspheres were confirmed by TGA, which resulted in higher conductivity (6.3 × 10^−5^ S/cm) than that of the pure PMMA microspheres (1.3 × 10^−13^ S/cm) [52]. Therefore, the selection of surfactants for dispersing nanotubes not only minimizes the impact of chemical modification on the inherent properties of the individual nanotube but also avoids the soluble problem of PMMA in organic solvents. Recently, snowman-like (SL) particles were coated with carboxylic acid-functionalized MWNTs (c-MWNTs) in the presence of CTAB using the same method [36] (Figure 3). The SL particles were prepared using a two-step process, in which cross-linked PMMA seeds were synthesized using a dispersion polymerization method (Figure 4a), and the SL particles were fabricated after a swelling process by seed polymerization (Figure 4b). The particles dispersed in the surfactant solution, which was carried out above the CMC (critical micelle concentration) to ensure that the entire surface of the SL particles could be coated with the surfactant during processing. Subsequently, the SL particles/surfactant solution was centrifuged to remove the redundant surfactant, which was unstable on the surface of the SL particles by centrifuge (Figure 4c). When the c-MWNTs were dispersed in the particle/surfactant solution, the hydrophobic tail groups of the CTAB could be adsorbed onto the particle surface and the hydrophilic head groups were adsorbed onto the c-MWNT by an electrostatic force to form the core-shell structured c-MWNT/SL particles. Therefore, the surfactant-assisted coating method is a simple and controllable process for fabricating core-shell structured ER materials. The conductivity could be adjusted by the content of c-MWNTs adsorbed on the surface of the particles, which increased significantly from 1.02 × 10^10^ S/cm (pure SL particles) to 4.06 × 10^−8^ and 6.10 × 10^−4^ S/cm using the 0.96 and 5.65 wt% MWNT coating, respectively. Comparative analysis of the dispersion and adsorption of MWNTs onto the SL particles with SDS was also reported [38]. The cationic surfactant CTAB is more effective than the anionic surfactant SDS for assisting the nanotubes adsorbed onto microparticles due to the presence of electrostatic interactions between the functionalized MWNTs and the surfactant, CTAB.

Ultrasonication instead of the use of a surfactant was also attempted to prepare homogenous MWNTs/PMMA dispersions, and is considered a simple method [39]. MWNTs (after acid treatment) with carboxylic acid functional groups were well dispersed in Di-water, and mixed with the PMMA colloid dispersion under sonication to form a homogenous suspension. The hydrophobic interaction between PMMA and the MWNTs also plays a dominate role in the system with the evaporation of distilled-water, which leads to the formation of a core-shell structure of c-MWNT/PMMA microspheres, exhibiting individual MWNTs instead of aggregated MWNTs (Figure 2c). The good dispersion and adsorption of MWNTs on the PMMA surface was attributed to ultrasonication, which can disrupt the MWNT aggregates during the violent collapse of cavitation bubbles in a few microseconds. The different adsorption states of MWNTs on the microspheres in different solvents are believed to be related to the degree of the CNTs dispersion in solution. The hydrogen bonding component of the Hansen solubility parameter [53] is believed to be the principal factor determining the dispersion states of the MWNTs in solvents because the nanotubes have carboxylic acid functional groups on the surface, which can help form hydrogen bonds between the c-MWNTs and solvent. Therefore, Di-water is a solution with a high hydrogen-bonding component that can lead to the good adsorption of MWNTs on the PMMA surface.

#### 2.1.2. Grafting-to Technique

A “grafting to” technique can also be used to fabricate core-shell-structured ER particles, in which the polymers chains bond covalently to the functional groups on the surface of the CNTs via special reactions, such as etherification and amidization [54,55]. Amino-functionalized MWNTs (MWNT-NH_2_) were attached successfully to the PMMA surface by covalent bonding between the amine groups on the MWNTs and carboxylic acid groups on PMMA [40]. In this method, MWNT-NH_2_ was prepared by chemical modification of the MWNT surface using hexamethylene diamine. SEM (Figure 2d) showed that the PMMA particles were well wrapped by MWNT-NH_2_ and the MWNT-NH_2_ was much shorter than c-MWNT (only after the acid treatment). This is because many defects formed on the MWNT-NH_2_ sidewalls during the functional process, which are easily broken into small fragments under ultrasonication.

### 2.2. Shish-Kebab Structured ER Particles

**Figure 5.**
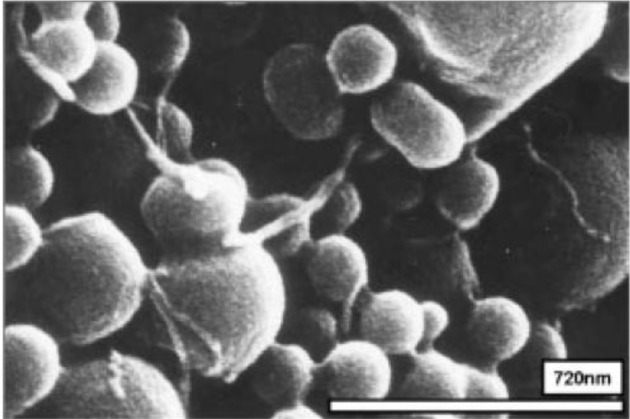
SEM image of MWNT/PMMA particles (reprinted with permission from [34], Copyright 2005 Wiley-VCH).

Park *et al.* [34] reported the polymerization of methyl methacrylate by suspension polymerization in the presence of MWNTs (after acid treatment) under sonication to obtain the MWNT/PMMA particles that displayed “shish-kebab”-shaped CNT/PMMA microspheres, as shown in Figure 5. Some MWNTs, like a bamboo spike, stabbed the PMMA microspheres, and others were attached to the surface of the spheres. This was attributed to the participation of MWNTs in a polymer reaction and the consumption of AIBN to form radicals to initiate the grafting of PMMA. To improve the dispersibility of the MWNTs and its compatibility with the particles, the MWNTs were dispersed not only in the monomer under sonication in advance but were also polymerized at 70 °C for 3 h with ultrasound. The electrical conductivity of the MWNTs/PMMA microspheres was 4.6 × 10^−11^ S/cm, as measured using a two-probe method with a pressed disc-type sample. The dielectric constant of the MWNT/PMMA particle-based ER fluid was 2.42 at 1 kHz using an impedance analyzer (4284A, Hewlett-Packard, Palo Alto, CA, USA).

### 2.3. Pearl Necklace Structured ER Particles

**Figure 6.**
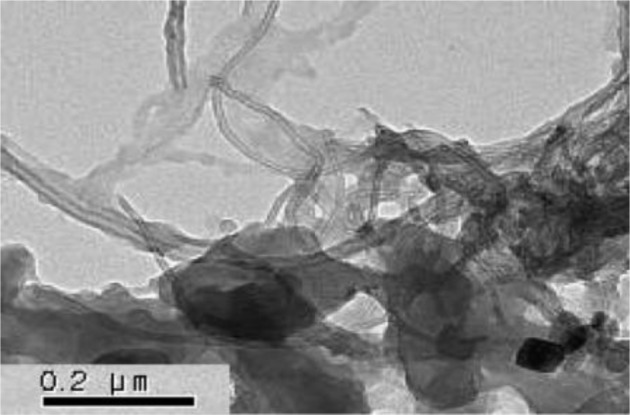
TEM image of the MWNT/PANI nanocomposite (reprinted with permission from [31], Copyright 2007 Elsevier).

PANI is a typical conducting polymer that is used widely as ER materials. Choi *et al.* [31] reported that the MWNT/PANI particles were prepared by oxidative dispersion polymerization using poly (vinyl alcohol) (PVA) as a surfactant to improve the dispersion ability of the MWNTs in water. The TEM image (Figure 6) of the synthesized MWNT/PMMA particles showed that the PANI is pierced with MWNT, giving it a “pearl necklace”-like structure. The effect of the PVA concentration was also examined, suggesting that 1 wt% PVA could disperse the MWNTs efficiently in water.

### 2.4. ER Particles with Other Structures

**Figure 7.**
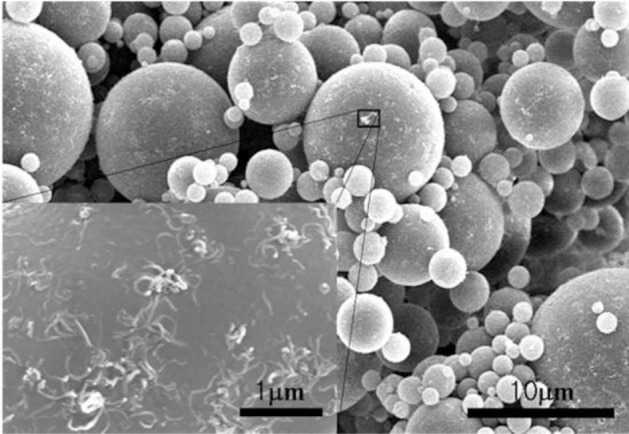
SEM morphology of MWNT/PMMA composite particles. The inset shows the surface morphology of the microspheres in detail (reprinted with permission from [33], Copyright 2007 Wiley-VCH).

**Figure 8.**
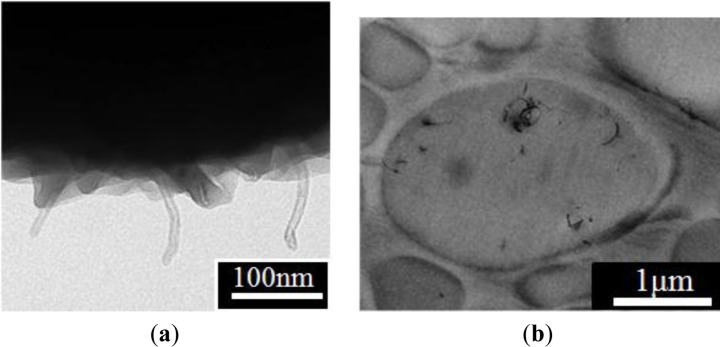
TEM image of (**a**) surface and (**b**) cross section of MWNT/PMMA composite particles (reprinted with permission from [33], Copyright 2007 Wiley-VCH).

Compared to the monodispersed PMMA microspheres, the MMA dispersion polymerization with the addition of chopped MWNTs exhibited polydispersed MWNT/PMMA microspheres [33], in which the diameter of the microbeads showed a broad distribution. This is believed to be relative to the size of the MWNTs and the number of individual MWNTs that participated in the formation of the MWNTs/PMMA microspheres. To obtain a homogenous dispersion of MWNTs, a steric stabilizer, poly(vinyl pyrrolidone) (PVP) was used to disperse the MWNTs in methanol. All ingredients were mixed under ultrasonication before polymerization. SEM (Figure 7) and TEM (Figure 8) confirmed that the MWNTs exist not only on the surface but also inside the PMMA particles. This suggests that the chopped MWNTs are short enough, which could enter the inside of particles when they are formed. MWNTs participate directly in the polymerization of the monomer. Therefore, the properties of the resulting composite particles are strongly dependent on the dispersion and size of the MWNTs. MWNTs have a shorter length than the diameter of the particles, which can exist both inside and outside the particles. On the other hand, they could pierce the microspheres to exhibit a “kebab-like” [34] or necklace structure [31] after polymerization.

## 3. ER Characteristics

**Figure 9.**
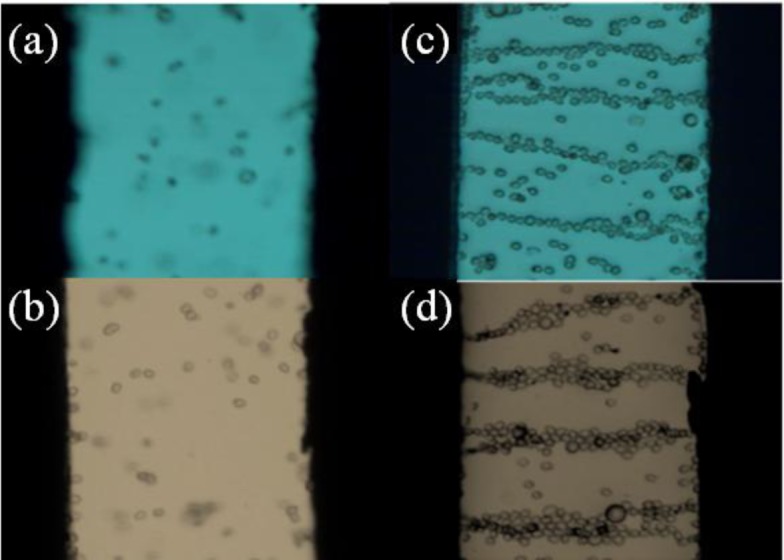
Optical microscopic images of the c-MWNT adsorbed SL particles using different surfactants dispersed in silicone oil (right) without an electric field [(**a**) by using CTAB; (**b**) by using SDS] and with an electric field of 0.44 kV/mm [(**c**) by using CTAB; (**d**) by using SDS) (reprinted with permission from [40], Copyright 2012 Elsevier).

For the core-shell-structured ER particles, they generally possess higher conductivity due to the dense coating of MWNTs on the particle surface. To avoid electric breakdown during the application of a high electric field, the ER behavior was observed by optical microscopy instead of a rheometer (MCR 300, Physica, Graz, Austria) under a low DC voltage (<1 kV/mm). The core-shell structured particle-based ER fluid was first prepared by dispersing the particles in silicone oil (generally with 10 vol%) and then treated under sonication to obtain a better dispersion. In the absence of an applied electric field, they dispersed randomly in silicone oil (Figure 9a,c), showing a liquid-like state. After applying an electric field, the particles shifted rapidly and connect to the neighboring particles to form a chain-like structure (Figure 9b,d) spinning towards the electrodes [10,23]. The structure remained stable as long as the field was applied.

The rheological properties of shear stress and shear viscosity as a function of a shear rate of the polymer/MWNT-based ER fluid with a small MWNT content were measured at (25 ± 0.1) °C using a rotational rheometer (MCR 300, Physica, Graz, Austria) quipped with a high voltage generator (HCP 14-12500, fug). The ER fluids were loaded in the cup of a concentric cylinder cell (CC17/E, inside cup diameter = 18.07 mm, gap size distance between cup and bob = 0.71 mm). The rotor (or bob) of the concentric cylinder was dipped in the ER fluid and drove by the motor of a rheometer. The ER fluids were prepared by dispersing the particles in silicone oil at different concentrations. To establish the equilibrium internal structure of the particles, an electric field was applied to the ER fluid for 3 min before the measurements. The flow curves for each ER fluid were obtained in controlled shear rate (CSR) mode.

**Figure 10.**
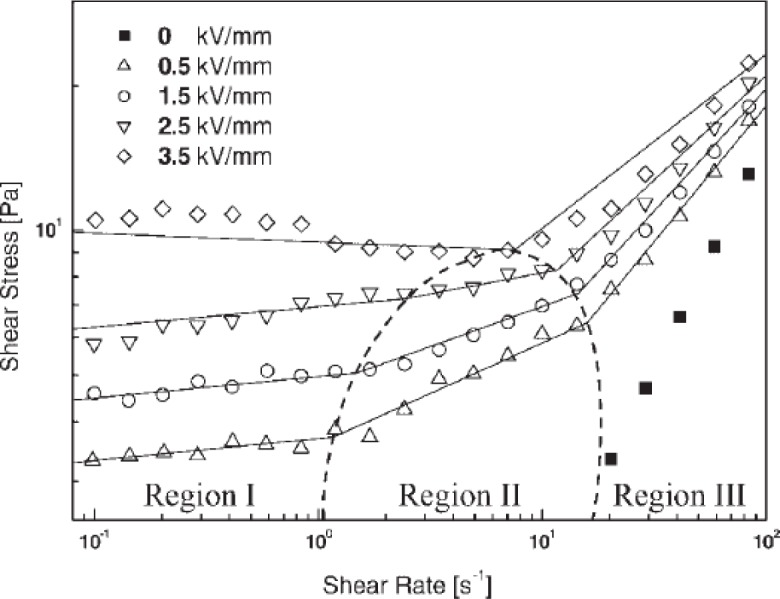
Shear stress *vs.* shear rate for MWNT/PMMA particle based ER fluid (reprinted with permission from [34], Copyright 2005 Wiley-VCH).

Figure 10 shows the flow curves of the shear stress *vs.* shear rate for the polymer/MWNT suspension [34] in silicone oil under a range of electric field strengths. When an electric field was applied to the suspension, it exhibited solid-like behavior in the low shear rate region with a non-vanishing yield stress, and the yield stress increased with the applied electric field strength because of the enhanced inter-particle interactions by increasing the degree of polarization of the MWNT/PMMA particles. This behavior indicated the ER fluids to be Bingham fluids, which is described by the Bingham model in Equation (1) with the τ*_y_* for the yield stress and η for the shear viscosity:

$$\{\begin{array}{cc} \tau =\eta \dot{\gamma }+\tau_{\text{y}} & \text{if }\tau \geq \tau_{y}\\ \dot{\gamma }=0 & \text{if }\tau <\tau_{y} \end{array}$$ (1)

where τ*_y_* is the yield stress and η is the shear viscosity. The MWNT/PMMA particles have enhanced interactions because the degree of polarization of particles increases with increasing applied electric field strength.

In addition, the flow curves of the MWNT based ER fluids under fixed electric field strengths were well described by the constitutive rheological equation, Cho-Choi-Jhon (CCJ) model (Equation (2)), along with other ER and MR fluid systems [33,36]:

$$\tau =\frac{\tau_{0}}{1+{\left(t_{1}\dot{\gamma }\right)}^{\alpha }}+\eta_{\infty }\left(1+\frac{1}{{\left(t_{2}\dot{\gamma }\right)}^{\beta }}\right)\dot{\gamma }$$ (2)

**Figure 11.**
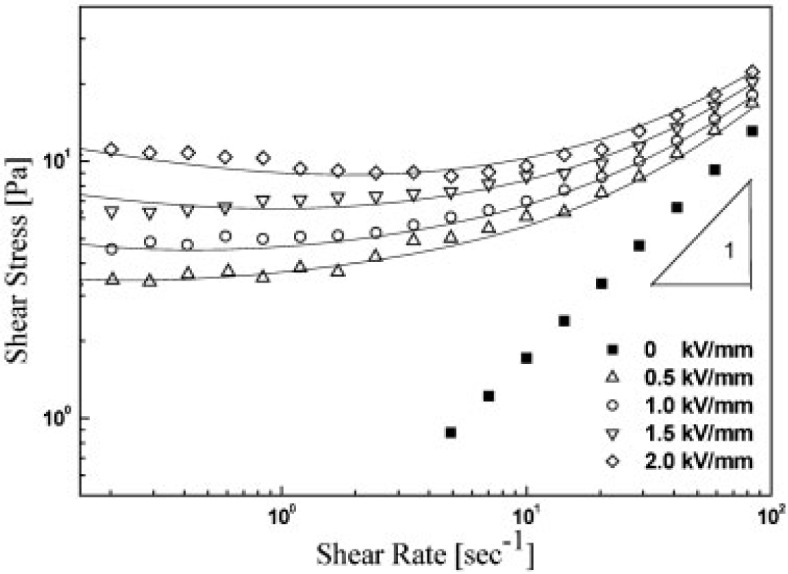
Shear stress *vs*. shear rate curve of MWNT/PMMA particle based ER fluid under various electric fields; the solid lines were obtained by CCJ model (reprinted with permission from [33], Copyright 2007 Wiley-VCH).

The CCJ model is a six-parameter model that is used widely to fit the experimental data for a range of ER suspensions [56]. Here, α is related to the decrease in the shear stress at a low shear rate region, and the exponent β ranges from 0 < β ≤ 1, because dτ/dγ ≥ 0 above the critical shear rate at which the shear stress becomes a minimum. t_1_ and t_2_ are the time constants in which *t*_1_ is considered to be the inverse of the shear rate at which the shear stress shows a minimum at a low shear rate region and *t*_2_ is related to the inverse of the shear rate at which pseudo-Newtonian behavior begins. η_∞_ is the viscosity at a high shear rate and is interpreted as the viscosity in the absence of an electric field. The first term in the equation implies shear stress behavior at a low shear rate region, particularly in the case of a decrease in the shear rate, and the second term describes the shear stress behavior well at a high shear rate region. The CCJ mode fits the shear stress in all shear rate regions well from the simple best-fitting method using the Excel program (despite six parameters, two values of yield stress and shear viscosity are easily estimated) and can provide a more accurate value for the systems of polymer/MWNT nanocomposites (Figure 11), particularly the data investigated at a relatively low shear rate. In contrast, the Bingham model could not describe the data accurately for the case of the decrease of shear stress [31,33,36].

**Figure 12.**
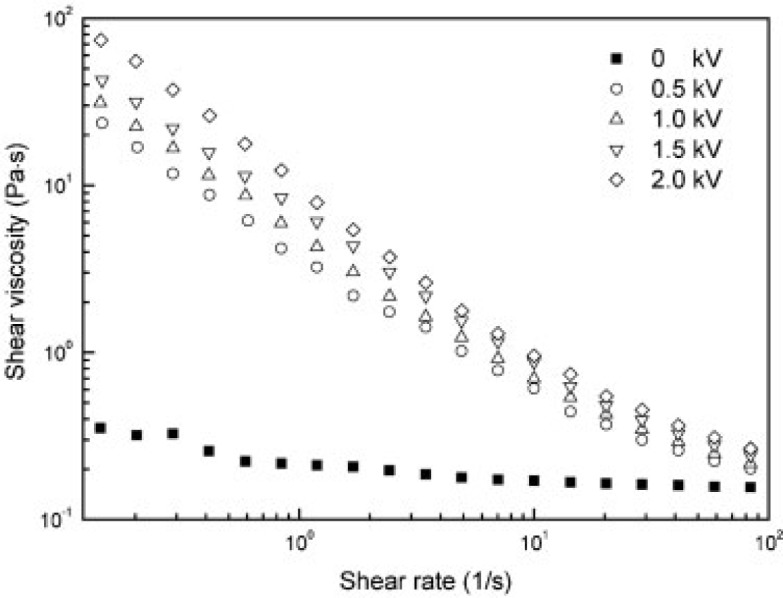
Flow curve of MWNT/PMMA particle based ER fluid under various electric fields (reprinted with permission from [33], Copyright 2007 Wiley-VCH).

In addition, the apparent shear viscosities of the polymer/MWNT-based ER fluid were also investigated at various electric field strengths as a function of the shear rate (Figure 12). The shear thinning behavior of these ER fluids increased with increasing electric field strength and the shear viscosity under the applied electric field was much higher than that under a zero electric field, particularly at low shear rates, suggesting that the nanocomposites became solid-like under an external electric field.

Other CNT-polymer nanocomposite based ER fluids [31,36,41,43] also display the similar trend as observed in Figures 10 and 12, *i.e*., the shear stress increases linearly with the shear rate (the slope is 1.0), showing a Newtonian behavior in absence of an electric field and the shear stress increases with increased electric field strength, exhibiting a Bingham fluid behavior. The zero shear viscosity under applied electric fields becomes much larger than that for the zero electric field applied. In addition, the shear thinning behavior is also observed to increase as the electric field strength increased. This rheological property is attributed to the formation of the polarization-induced fibrillar or columnar structure of the dispersed particles under applied electric fields.

## 4. Conclusions

MWNTs by virtue of the functional groups on their surface or by the help of surfactants were dispersed well in some organic solvents and participated in polymerization under a sonication treatment to form polymer/MWNT nanocomposites, in which its structure is strongly dependent on the type of the CNT and fabrication processing. The polymer/MWNT nanocomposites exhibited a superior ER response under an applied electric field, suggesting that the nanocomposites show a higher conductivity and dielectric constant than the pure particles due to the participation of the MWNTs.
